# Consumer Satisfaction about Hospital Services: A Study from the Outpatient Department of a Private Medical College Hospital at Mangalore

**DOI:** 10.4103/0970-0218.51220

**Published:** 2009-04

**Authors:** KS Prasanna, MA Bashith, S Sucharitha

**Affiliations:** Department of Community Medicine, Father Muller Medical College, Mangalore, India

**Keywords:** Consumer satisfaction, hospital services, clinical care

## Abstract

**Background:**

Consumer satisfaction is an important parameter for assessing the quality of patient care services. There is a need to assess the health care systems regarding the consumer satisfaction as often as possible.

**Objectives:**

To assess the consumer satisfaction regarding the services provided in our outpatient department in terms of clinical care, availability of services, waiting time, and cost.

**Materials and Methods:**

A 27-item pre-tested questionnaire was given to 100 patients (caretakers in pediatric patients) at the end of their O.P.D visit from 3 to 4 pm for 5 days from November 7, 2005 to November 11, 2005. The items in the questionnaire referred to particulars of the patients such as age, sex, occupation, department requested, lab, and medical stores. While analyzing, they were grouped into categories like availability, clinical care, waiting time, and cost. The responses were expressed in proportions.

**Results:**

The availability of services and clinical care was found to be satisfactory. 81% of the respondents found the communication by the doctor good, 97% of the respondents were satisfied about the explanation of the disease by the doctor The average time required for consulting the doctor was 46.5 ± 20.9 min. But when time spent in pharmacy was considered, it was not significantly satisfactory. The cost of investigation was significantly moderate or high in 97% of the respondents.

**Conclusions:**

Recommendations are required for reduction of time spent in the pharmacy and the cost of investigations to improve consumer satisfaction.

## Introduction

Health care has two connotations (a) health care programs and (b) medical care organizations. Medical care organizations are mainly providing curative care. They are attractive and high-tech oriented and they should be cost effective.(1) In recent years, quality assurance has emerged as an internationally important aspect in the provision of health care services.(2) The health care system depends on availability, affordability, efficiency, feasibility, and other factors.(3) Consumer satisfaction is recognized as an important parameter for assessing the quality of patient care services. Satisfaction regarding the attitude of providers toward these services is expected to affect treatment outcome and prognosis. There is a need to analyze the health care system as often as possible.(4)

Consumer satisfaction regarding medical care organizations like our tertiary care hospital is important in the provision of services to patients. So, we have designed a study to assess consumer satisfaction with regard to clinical care such as the approach of the doctor, examination, education on taking medication, availability of services, waiting time, and cost provided in the outpatient department of our medical college hospital.

## Materials and Methods

The study was carried out in the outpatient department of Father Muller Medical College Hospital, Mangalore. A 27 item pre-tested and pre-structured questionnaire was given to the patients or their attendants at the end of their outpatient visit. A total of 100 patients were selected at random in a time span of 5 days from November 7, 2005 to November 11, 2005 between 3 and 4 p.m. The items in the questionnaire referred to the particulars of the patient such as age, sex, occupation, The concerned department, service particulars in the registration counter, concerned doctor in the respective department, the lab, and the medical stores. The questionnaire included choices like convenient/inconvenient, satisfactory/unsatisfactory, easy/difficult, good/moderate, adequate/inadequate, and 30 minute time slots of actual time spent in each stage of the visit. Informed consent was obtained from the patient. The patients were told that the purpose of the study was to assess the consumer satisfaction of services provided by the hospital so as to bring about further improvement of services. The patients were also told that the investigator was not part of the treatment team. It was also emphasized that they were free to give their honest responses. Anonymity of the examining doctor and the patient was maintained. The responses were expressed in proportions.

## Results

The study population consisted of 100 patients (66 males and 34 females). A total of 65% were in the age group between 15-45 years old [Table 1]. Respondents were patients themselves (90%) and accompanying relatives for pediatric patients younger than 15 years old (10%). The opinions of the patients were grouped into the following 4 groups:

**Table 1:** Distribution of the respondents according to the age sex, occupation and the concerned departments

	Total number of respondents (n=100)
Age	
15-29	34
30-44	31
45-59	19
60 and above	16
Sex	
Male	66
Female	34
Occupation	
Skilled	28
Unskilled	29
Unemployed	9
Housewife	21
Student	13
Department	
Medicine	26
Surgery	14
Ob. and gynecology	12
Pediatrics	6
Orthopedics	12
Dermatology	4
ENT	18
Ophthalmology	8

1. Availability
2. Clinical care
3. Waiting time
4. Cost

Overall availability of services [Table 2] was very good regarding the seating arrangements in the outpatient department (100%) and the cleanliness of the outpatient department (100%). A total of 98% of the respondents were satisfied with the outpatient department timings, 88% of the respondents were satisfied with the services of the outpatient nursing staff, 84% of the respondents found it easy to locate the concerned specialist department in the outpatient department, and 99% of the respondents found the availability of the doctors in the outpatient department to be adequate. Only 14% of the respondents found it difficult to locate the pharmacy.

**Table 2:** Distribution of responses from the respondents according to availability of services

Availability	Total no. (n=100)
Seating arrangement in OPD	
Satisfactory	100
Unsatisfactory	00
Cleanliness of the OPD	
Satisfactory	100
Unsatisfactory	00
OPD Timings	
Satisfactory	98
Unsatisfactory	2
Services by the paramedical staff (OPD nurses)	
Satisfactory	88
Unsatisfactory	12
Finding the concerned specialist department in the OPD	
Easy	84
Difficult	16
Availability of the doctor in the OPD	
Adequate	99
Inadequate	1
Finding the pharmacy	
Easy	13
Satisfactory	73
Unsatisfactory	14

Regarding clinical care [Table 3], 97% of the respondents found the approach of the doctors satisfactory, 81% of the respondents found the communication by the doctor good, 97% of the respondents were satisfied about the explanation of the disease by the doctor, 94% of the respondents found clinical care satisfactory, and 97% found the doctor efficient. A total of 99% of the respondents gave an opinion that the investigations were conducted necessarily and 83% of the respondents were satisfied with the number of investigations that are necessary. Interpretation of the investigation report by the doctor to the patient was satisfactory in 96% of the respondents. The nature of prescription was either simple and easy or satisfactory in 83 % of the respondents. Instruction regarding medication usage by dispensing pharmacists was satisfactory in 86% of the respondents.

**Table 3:** Distribution of responses from the respondents regarding clinical care

Clinical care	Total no. (n=100)
Approach by the doctor	
Satisfactory	97
Unsatisfactory	3
Communication by the doctor	
Good	81
Moderate	19
Explanation about the disease to the patient	
Satisfactory	97
Unsatisfactory	3
Clinical care	
Satisfactory	94
Unsatisfactory	6
Efficiency	
Satisfactory	97
Unsatisfactory	3
Opinion about the need of investigation as assessed by the patient	
Necessary	99
Unnecessary	1
Opinion about the number of investigations as assessed by the patient	
Necessary	83
Unnecessary	17
Interpretation of the investigation report by the doctor to the patient	
Satisfactory	96
Unsatisfactory	4
Nature of the prescription	
Simple and easy	8
Satisfactory	75
Complex and difficult	17
Instructions for taking medication by dispensing pharmacists	
Satisfactory	86
Unsatisfactory	10
Not given	14

With regard to waiting time [Table 4], 79% of the patients found that the time required for registration was convenient for them (average time = 12.6 ± 6.87 min.). A total of 89% of the patients found the concerned department conveniently (average time = 10.05 ± 3.99 min.). The time required for consulting the doctor was less than 30 minutes in 20% of the cases, 30 to 60 minutes in 57% of the cases, 60 to 90 minutes 21% of the cases, and 90 to 120 minutes in 2% of the cases (average time = 46.5 ± 20.9 min.). Time taken for the investigations was satisfactory in 72% of the patients (average time = 142.75% ± 234.92 min). Time required to locate the pharmacy was satisfactory in 91% of the cases (average time = 9.9% ± 3.01 min) and the time spent in the pharmacy was satisfactory in only 53% of the patients (average time = 26.8 ± 18.36 min.).

**Table 4:** Distribution of responses of the respondents regarding waiting time

Waiting time	Total no. (n=100)	Mean±S.D in min
Time gap between coming to the OPD and getting registered		
Convenient	79	12.6±6.87
Inconvenient	21	
Time required to find the concerned department		
Convenient	89	10.05±3.99
Inconvenient	11	
Time required to consult the doctor		
< ½ hr	20	46.5±20.9
½ - 1 hr	57	
1 and 1 ½ hr	21	
1 and ½ hr - 2 hr	2	
Time taken for investigation		
Satisfactory	72	142.75±234.92
Unsatisfactory	28	
Time required to locate the pharmacy		
Satisfactory	91	9.9±3.01
Unsatisfactory	9	
Time spent in pharmacy		
Satisfactory	53	26.8±18.36
Unsatisfactory	47	

The cost of registration and consultation [Table 5] was satisfactory for 100% of the respondents. The cost of investigation was low for 3% of the respondents, moderate for 73% of the respondents, and high for 24% of the respondents. The cost of medicines was satisfactory for 80% of the respondents. This may be dependent on the consumer's purchasing power and different costs of medicines.

**Table 5:** Distribution of responses from the respondents regarding cost

Cost	Total no. (n=100)
Cost of registration	
Satisfactory	100
Unsatisfactory	00
Cost of consultation fees of the doctor	
Satisfactory	100
Unsatisfactory	00
Cost of investigation	
Low	3
Moderate	73
High	24
Cost of medicines	
Satisfactory	80
Unsatisfactory	20

## Discussion

In this study, each step of the outpatient services assessment was made by the consumer on factors such as availability of services, clinical care, time, and cost of services. It was found to be satisfactory regarding the availability of services and clinical care. But when the time spent in pharmacy was analyzed, it was considered that it was not significantly satisfactory. The costs of investigation were significantly moderate and high in 97% of the cases as assessed by the respondents. In a study by Acharya and Acharya, 82.8% of the respondents showed the approach of the doctor is personal. In their study, 81.6% regarded the explanation of the disease to the patient as satisfactory, 93.2% of the subjects were satisfied with the examination by the doctor, and it was simple and easy to understand in 60% of the cases.^(5)^

## Conclusions

According to the consumer's opinion, the study showed good results with respect to availability and clinical care. Recommendations regarding ways to reduce the time spent in the pharmacy and the cost of investigations are required to improve consumer satisfaction.
